# Supplementary material for: Spatial variations and determinants of malnutrition among under-five children in Nigeria: A population-based cross-sectional study
Source: PLoS One. 2023 Apr 11;18(4):e0284270. doi: 10.1371/journal.pone.0284270 (PMC10089328; doi:10.1371/journal.pone.0284270)
Supplement: Attachment — Submitted filename: response to comments.docx [file pone.0284270.s002.docx]

PONE-D-22-26344R1

**Spatial variations and Determinants of malnutrition Among Under-five Children in Nigeria: A population-based cross-sectional study**

Dear Editor,

Thank you for the invitation to submit a revised version of our manuscript.

We have included the following items:

- A rebuttal letter that responds to each point raised by the academic editor and reviewer(s).
- A marked-up copy of our manuscript that highlights changes made to the original version. However, we have left out the changes which were necessary due to the PLOS formatting guidelines. This because of readability.
- An unmarked version of our revised paper without tracked changes.
- The minimal data set underlying the results described in our manuscript has been uploaded as a supporting information (SI) file

Below you will find our answers to the raised issues and questions.

**Journal Requirements:**

1. *Please ensure that your manuscript meets PLOS ONE's style requirements, including those for file naming.*

**Response:** This is duly noted.

2. *In your Data Availability statement, you have not specified where the minimal data set underlying the results described in your manuscript can be found. PLOS defines a study's minimal data set as the underlying data used to reach the conclusions drawn in the manuscript and any additional data required to replicate the reported study findings in their entirety. All PLOS journals require that the minimal data set be made fully available. For more information about our data policy, please see http://journals.plos.org/plosone/s/data-availability.*

*"Upon re-submitting your revised manuscript, please upload your study’s minimal underlying data set as either Supporting Information files or to a stable, public repository and include the relevant URLs, DOIs, or accession numbers within your revised cover letter. For a list of acceptable repositories, please see http://journals.plos.org/plosone/s/data-availability#loc-recommended-repositories. Any potentially identifying patient information must be fully anonymized.*

*We will update your Data Availability statement to reflect the information you provide in your cover letter.*

**Response:** Thank you. The minimal data set underlying the results described in our manuscript has been uploaded as a supporting information (SI) file.

3. *Your ethics statement should only appear in the Methods section of your manuscript. If your ethics statement is written in any section besides the Methods, please move it to the Methods section and delete it from any other section. Please ensure that your ethics statement is included in your manuscript, as the ethics statement entered into the online submission form will not be published alongside your manuscript.*

**Response:** Ethics statement has now been moved to the Methods section of the manuscript.

4. *We note that Figures 1, 3 and 5 in your submission contain [map/satellite] images which may be copyrighted. All PLOS content is published under the Creative Commons Attribution License (CC BY 4.0), which means that the manuscript, images, and Supporting Information files will be freely available online, and any third party is permitted to access, download, copy, distribute, and use these materials in any way, even commercially, with proper attribution. For these reasons, we cannot publish previously copyrighted maps or satellite images created using proprietary data, such as Google software (Google Maps, Street View, and Earth). For more information, see our copyright guidelines: http://journals.plos.org/plosone/s/licenses-and-copyright.*

*We require you to either (1) present written permission from the copyright holder to publish these figures specifically under the CC BY 4.0 license, or (2) remove the figures from your submission:*

**Response:** Truly, **Figure 1** may be copyrighted, and we have thus removed it from the submission. However, we can confirm that **Figures 3 and 5** were created or constructed by the authors based on the study data as part of the spatial data analysis using R (version 4.2.1) package. Permission from a copyright holder is not applicable in this context.

**Reviewers’ comments**

We would like to thank the reviewers for their comments as well as the opportunity to revise our manuscript for further consideration in your journal. We have addressed all the comments from the reviewers and below we provide a detailed description of the revisions made. In the revised manuscript, the green-shaded texts indicate changes made in response to suggestions and questions of the reviewers.

**Reviewer #1**

***Comment:*** *This paper has great potential and would be relevant to Nigeria if all amendments are given revised. The authors show great composure, especially in the methods and presentation of the results.*

*Material and methods:*

*Please use the following format for this section*

*Data management: [How to manage data]*

*Descriptive statistics: [How to summarize data]*

*Explorative statistics: [ inferential statistics you have used]*

*Results:*

*Please use the following format for this section*

*General characteristics: [status of the outcome variable, summarize the data]*

*Factors associated with outcome:*

**Response:** The suggested presentation format is highly appreciated. We have implemented as suggested. However, your suggested format for the results section “General characteristics” has already been implied in the descriptive statistics section suggested under the material and methods section.

**Reviewer #2**

*Thank you so much for requesting me to review this manuscript. The manuscript assessed the “Spatial variations and determinants of malnutrition among under five children in Nigeria: A population-based cross-sectional study”.*

*The manuscript is interesting. In general, it is well done but there are some comments that need to be fixed.*

*Introduction*

***Comment:*** *1. Line 39: “ … from 6.6million …..2017” Reference(s) is needed.*

**Response:** Thank you. The requested reference has now been cited.

*Study variables*

***Comment:*** *Table2. I wonder why you did not consider the following variables:*

*immunization card*

*History of immunization.*

**Response:** We agree that these are important variables, and many more of such factors that potentially affect malnutrition; however, we are limited by the available variables in the NDHS database. Data on the suggested variables were not collected.

***Comment:*** *2. Line 93: Table1: I think you need to provide source and to test for the reliability of the index.*

**Response:** Though the CIAF index has been referenced in lines 57 and 58, we have now referenced the original source of the CIAF (Nandy & Svedberg, 2000) close to Table 1 in line 104.

*Specification of Bayesian prior distribution and hyper-parameters*

***Comment:****3. Line159: “… and diffuse priors…..( you Wrote beta r1 or beta r1) and…this is confusing, is there any difference between them.*

**Response:** The sentence is truly confusing. We have now rephrased **(see lines 161,162).**

***Comment:*** *4. Did you consider possible interaction effect(s)?*

**Response:** In the final analysis, no, we did not. In a preliminary analysis, the inclusion of interaction effects worsened our model fit, hence the need to drop them from the main analysis.

***Comment:*** *5. I think mother’s nutrition knowledge may also be an important factor and it is not considered in this research. Why?*

**Response:** We agree that mother’s nutrition knowledge is a good potential predictor variable in this study; however, we are limited by the available variables in the NDHS database. Data on the suggested variable were not collected.

***Comment:*** *6. Line 334: … larger the family, the less per capita input” It is not clear please explain it.*

**Response:** Thank you, we have rephrased the sentence to read better **(See lines 339, 340).** We intended to imply the dilution model which predicts that larger families reduce the amount of resources (time, energy, money) available for each child, thus hindering social and physical development.

***Comment:*** *7. Line 257: …48 moths, after which the effect decreased…” This interpretation is correct. Please observe the figure and interpret it correctly.*

**Response:** Thanks for observing this interpretation error. We have now revised the interpretation.

Sincerely,

Lateef Amusa (PhD), (on behalf of all the authors)
